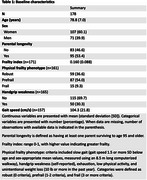# Microstructural Integrity of the Hypothalamus and Amygdala Relates to Distinct Components of Frailty

**DOI:** 10.1002/alz70861_108208

**Published:** 2025-12-23

**Authors:** Katherine Wang, Roman Fleysher, Sanish Sathyan, Tina Gao, Erica Weiss, Rachel Shornick, Joe Verghese, Michael L. Lipton, Nir Barzilai, Sofiya Milman, Sandra Aleksic

**Affiliations:** ^1^ Albert Einstein College of Medicine, Bronx, NY USA; ^2^ Columbia University Irving Medical Center, New York, NY USA; ^3^ Stony Brook University, Stony Brook, NY USA; ^4^ Montefiore Medical Center, Bronx, NY USA

## Abstract

**Background:**

Frailty, an aging‐related syndrome marked by diminished physiologic reserve and impaired homeostasis, increases risk for cognitive impairment and dementia; however, its neurobiology remains incompletely characterized. Neuroimaging studies link frailty to small vessel pathology and white matter microstructural alterations, but its relationship with subcortical gray matter (GM) microstructure is underexplored. This study examined associations between frailty and diffusion tensor imaging (DTI)–derived microstructural integrity in subcortical GM, including the hypothalamus—a key homeostatic hub potentially involved in aging‐related cognitive vulnerability.

**Method:**

Older adults from the LonGenity (*n* =178; Table 1), half with long‐lived parents, underwent baseline brain magnetic resonance imaging (MRI) and annual frailty assessments using both the cumulative deficits frailty index and the physical frailty phenotype. Generalized linear mixed models tested associations between frailty and DTI metrics—bilateral volume‐weighted mean diffusivity (MD) and fractional anisotropy (FA)—in the hypothalamus and six other subcortical GM regions: hippocampus, amygdala, thalamus, accumbens, caudate, and putamen. Greater MD and lower FA may indicate poorer microstructural integrity. Models adjusted for age, time, sex, parental longevity, and intracranial volume. Exploratory analyses examined associations between MD/FA and physical function (handgrip strength, gait speed). Frailty index was square root–transformed; Bonferroni correction addressed multiple comparisons.

**Result:**

Participants completed an average of two frailty assessments, totaling 373 frailty index and 347 physical frailty evaluations. Hypothalamic FA was negatively associated with the frailty index (beta [95% confidence interval, CI]: −0.022 [−0.038, −0.006] per 1 standard deviation [SD] of FA, *p* =0.006, Bonferroni–*p*=0.042); frailty index showed no significant associations with other regions’ FA or any MD. Physical frailty and handgrip strength were unrelated to MD or FA, but slower gait correlated with higher MD in the hypothalamus, hippocampus, and amygdala (beta [95% CI] cm/s per 1 SD of MD, *p* ‐value, respectively: −3.7 [−6.8, −0.5], 0.02; −3.8 [−7.7, 0.04], 0.049; −5.9 [−9.2, −2.7], 0.0004), although only amygdala MD remained significant after Bonferroni correction (*p* =0.003).

**Conclusion:**

These findings implicate hypothalamus in cumulative deficits frailty and amygdala in slower gait speed in older adults. Subcortical gray matter, particularly regions involved in homeostatic and emotional regulation, may play a role in the neurobiology of frailty.